# Standardized Prevalence Ratios for Atrial Fibrillation in Adult Dialysis Patients in Japan

**DOI:** 10.2188/jea.JE20150077

**Published:** 2016-05-05

**Authors:** Masaki Ohsawa, Kozo Tanno, Tomonori Okamura, Yuki Yonekura, Karen Kato, Yosuke Fujishima, Wataru Obara, Takaya Abe, Kazuyoshi Itai, Kuniaki Ogasawara, Shinichi Omama, Tanvir Chowdhury Turin, Naomi Miyamatsu, Yasuhiro Ishibashi, Yoshihiro Morino, Tomonori Itoh, Toshiyuki Onoda, Toru Kuribayashi, Shinji Makita, Yuki Yoshida, Motoyuki Nakamura, Fumitaka Tanaka, Mutsuko Ohta, Kiyomi Sakata, Akira Okayama

**Affiliations:** 1 Department of Hygiene and Preventive Medicine, Iwate Medical University, Yahaba-cho, Iwate, Japan; 1 岩手医科大学統合基礎講座衛生学公衆衛生学講座; 2 Department of Preventive Medicine and Public Health, Keio University, Tokyo, Japan; 2 慶応大学医学部衛生学公衆衛生学講座; 3 Division of Nephrology, Iwate Prefectural Central Hospital, Morioka, Japan; 3 岩手県立中央病院内科腎臓内科分野; 4 Department of Urology, Iwate Medical University, Morioka, Japan; 4 岩手医科大学医学部泌尿器科学講座; 5 Department of Nutritional Sciences, Morioka University, Takizawa, Iwate, Japan; 5 盛岡大学栄養科学部栄養科学科; 6 Department of Neurosurgery, Iwate Medical University, Morioka, Japan; 6 岩手医科大学医学部脳神経外科学講座; 7 Department of Community Health Sciences, University of Calgary, Calgary, Canada; 7 カルガリー大学地域保健学部; 8 Department of Clinical Nursing, Shiga University of Medical Science, Otsu, Japan; 8 滋賀医科大学医学部臨床看護学講座; 9 Department of Internal Medicine, Iwate Medical University, Morioka, Japan; 9 岩手医科大学医学部内科学講座; 10 Department of Health and Physical Education, Faculty of Education, Iwate University, Morioka, Japan; 10 岩手大学教育学部保健体育科; 11 Iwate Health Service Association, Morioka, Japan; 11 岩手県予防医学協会; 12 The Research Institute of Strategy for Prevention, Tokyo, Japan; 12 生活習慣病予防研究センター

**Keywords:** atrial fibrillation, end-stage renal disease, standardized prevalence ratio, 心房細動, 末期腎不全患者, 標準化有病比

## Abstract

**Background:**

While it is assumed that dialysis patients in Japan have a higher prevalence of atrial fibrillation (AF) than the general population, the magnitude of this difference is not known.

**Methods:**

Standardized prevalence ratios (SPRs) for AF in dialysis patients (*n* = 1510) were calculated compared to data from the general population (*n* = 26 454) living in the same area.

**Results:**

The prevalences of AF were 3.8% and 1.6% in dialysis patients and the general population, respectively. In male subjects, these respective values were 4.9% and 3.3%, and in female subjects they were 1.6% and 0.6%. The SPRs for AF were 2.53 (95% confidence interval [CI], 1.88–3.19) in all dialysis patients, 1.80 (95% CI, 1.30–2.29) in male dialysis patients, and 2.13 (95% CI, 0.66–3.61) in female dialysis patients.

**Conclusions:**

The prevalence of AF in dialysis patients was twice that in the population-based controls. Since AF strongly contributes to a higher risk of cardiovascular mortality and morbidity in the general population, further longitudinal studies should be conducted regarding the risk of several outcomes attributable to AF among Japanese dialysis patients.

## 抄録

**背景:**

日本人透析患者の心房細動有病率は一般住民の心房細動有病率と比べて高いことが想定されるが、 実際には明らかにされていない。

**方法:**

地域悉皆的コホート研究に参加した透析患者1,510名を解析対象として、同一地域に住む一般住民26,454名を基準とした透析患者の心房細動標準化有病比 (SPR) を算出した。

**結果:**

透析患者集団の心房細動有病率は3.8% で一般集団では1.6% だった。男性では心房細動有病率がそれぞれ4.9% と 3.3%、女性では1.6% と 0.6%だった。標準化有病比（95%信頼区間）は男女全体で2.53 (1.88–3.19) 、男性で 1.80 (1.30–2.29) 、女性で 2.13 (0.66–3.61) だった。

**結論:**

地域ベースの集団を対象とした解析結果では、日本人透析患者集団では一般集団に比べて心房細動有病率が2倍高かった。心房細動は心血管疾患死亡や罹患に強く影響するリスク要因であり、心房細動が日本人透析患者の予後にどのような影響を与えているのかを縦断研究で明らかにすることが望まれる。

## INTRODUCTION

Patients with end-stage renal disease (ESRD) have extremely high mortality rates, and the leading cause of death among ESRD patients is cardiovascular disease.^1^ These patients have been shown to have high incidence and prevalence rates of atrial fibrillation (AF).^2^ AF also increases the risk of all-cause death and incident stroke in patients with ESRD.^2^ Markedly lower mortality rates have been reported in dialysis patients in Japan than in dialysis patients in other countries,^3^ and treatment regimens developed in Western countries should not be implemented in Japan without modifications, due to the very different characteristics and backgrounds of the patient populations. We should establish preventive measures for improving the prognosis of Japanese dialysis patients with AF using epidemiological evidence based on this specific population.

However, such data are currently insufficient. Basic information is needed regarding AF in Japanese dialysis patients, such as its prevalence, as well as AF-related outcome risks, including death, stroke, heart failure, and medical and social burdens. In this study, we used a population-based survey to determine the prevalence of AF in both dialysis patients and community dwellers living in the same area.

## METHODS

### Subjects

The study subjects included participants in both the KAREN Study (dialysis patients) and the Iwate-KENCO Study (general population). The study region consisted of the northern part of Iwate Prefecture. The methodologies of the KAREN Study^4^ and the Iwate-KENCO Study^5^ have been described elsewhere. In the KAREN Study, the original cohort study sample consisted of 1214 dialysis patients enrolled in 2005, in addition to 287 patients recruited in 2006 and 128 patients recruited in 2007. Of these 1629 dialysis patients, we excluded 119 due to a lack of electrocardiographic data. We used data from 1510 dialysis patients, of whom 57 had AF. In the Iwate-KENCO Study, the original cohort study consisted of 26 469 participants. We excluded subjects that were less than 20 years of age (*n* = 15). We ultimately analyzed data from 26 454 participants, of whom 411 had AF. The studies were approved by the Medical Ethics Committee of Iwate Medical University and conducted in accordance with the Declaration of Helsinki.^4^^,^^5^

### Measurements

The data gathering methodology has been previously described.^4^^,^^5^ Hypertension (HT) was defined as systolic blood pressure (SBP) of 140 mm Hg or higher, diastolic blood pressure of 90 mm Hg or higher, use of antihypertensive agents, or a combination of these. Diabetes (DM) was defined as plasma glucose level of 200 mg/dL or higher, plasma HbA_1c_ level (NSGP equivalent value) of 6.5% or higher, use of anti-diabetes agents, or a combination of these. Dyslipidemia was defined as serum total cholesterol (TC) level of 220 mg/dL or higher, serum high density lipoprotein cholesterol (HDL-C) level of less than 40 mg/dL, use of anti-hyperlipidemia agents, or a combination of these. Regular alcohol drinking was defined as drinking five days or more per week.

In the KAREN Study, a resting 12-lead electrocardiogram (ECG) recorded close to the day of the initial survey at the dialysis institute was collected from each patient. Three medical doctors independently evaluated the ECG findings and identified cases of prevalent AF (including paroxysmal AF and atrial flutter). In cases of inconsistent evaluations, final judgments were made after deliberations at the approval meeting. In the Iwate KENCO Study, the baseline survey included a resting 12-lead ECG performed in each participant after 5 minutes of rest. A trained clinical technician and a medical doctor in the Iwate Health Service Association independently evaluated the ECG findings according to the original coding system.^5^ Prevalent cases of AF were determined based on the presence of chronic or paroxysmal AF/flutter.

### Statistical analysis

Characteristics of dialysis patients and community dwellers were shown by providing mean age (standard deviation [SD]) and age-adjusted means (95% confidence intervals [CIs]) of body mass index (BMI), SBP, serum TC level, serum HDL-C level, and glycosylated hemoglobin (HbA_1c_) level using analysis of covariance (ANCOVA). High-sensitivity C-reactive protein (hsCRP) level was expressed as age-adjusted geometric mean (95% CI). The age-adjusted prevalence of each factor (AF, overweight, HT, DM, dyslipidemia, current smoking, past smoking, and regular drinking) was estimated using logistic regression analysis. Age adjustment was performed with a 65-year-old person as the reference using analysis of ANCOVA or logistic regression.

Since we had a dataset of dialysis patients with a relatively small sample size, we performed an indirect age-standardization for the comparison of prevalence of AF between dialysis patients and community dwellers to avoid distortion resulting from inappropriate age-adjustment when data are sparse. To estimate standardized prevalence ratio (SPR; ratio of observed prevalence to expected prevalence) of AF in dialysis patients, we first determined age-stratified prevalence of AF in community dwellers. Stratum-specific prevalence rate of AF in each age category among community dwellers was multiplied by the number of subjects in the corresponding age category in dialysis patients, and then the expected number of subjects with AF in each age category among dialysis patients was estimated and the sum of expected numbers of AF subjects in age categories was calculated (ie, the expected prevalence of AF in dialysis patients). The 95% CI of SPR was estimated as follows: SPR ± 1.96 √(SPR/expected number).^6^

## RESULTS

Table 1 shows characteristics of participants stratified by sex in the dialysis patients and community dwellers. The mean age of community-dwelling male subjects was 2 years older than the mean ages in the other three groups. Age-adjusted mean levels of BMI, TC, HDL-C, and HbA_1c_ in dialysis patients were lower than those in community dwellers for both male and female subjects. Age-adjusted mean levels of SBP and hsCRP in dialysis patients were higher than the mean levels in community dwellers. Age-adjusted prevalence rates of AF were 4.5% in male dialysis patients and 1.4% in female dialysis patients, and these rates were 1.67-fold and 2.33-fold higher than the adjusted prevalence rates in community dwellers (*P* < 0.05 by logistic regression). Adjusted prevalence rates of HT and DM in dialysis patients were higher than the rates in community dwellers for both male and female subjects (*P* < 0.05 by logistic regression).

**Table 1.** Characteristics of participants stratified by sex both in community dwellers and dialysis patients

Subjects		Community dwellers (*n* = 26 454)		Dialysis patients (*n* = 1510)	
	
		Men	Women	Men	Women
		(*n* = 9155)	(*n* = 17 299)	(*n* = 1003)	(*n* = 507)
Mean (SD) age	years	64.3 (11.4)	61.6 (11.5)	61.8 (12.9)	62.4 (12.8)
Age-adjusted mean (95% CI)					
BMI	kg/m^2^	23.9 (23.8–23.9)	24.1 (24.0–24.1)	21.3 (21.1–21.5)^a^	20.3 (20.0–20.6)^a^
SBP	mm Hg	131.0 (130.6–131.4)	127.0 (126.8–127.3)	153.6 (152.0–155.1)^a^	153.4 (151.2–155.6)^a^
TC	mg/dL	191.3 (190.6–192.0)	205.8 (205.3–206.3)	147.0 (144.8–149.2)^a^	164.0 (161.0–167.1)^a^
HDL-C	mg/dL	55.9 (55.6–56.2)	60.9 (60.7–61.1)	44.8 (43.9–46.0)^a^	50.3 (49.0–51.6)^a^
HbA_1c_	%	5.55 (5.54–5.56)	5.53 (5.52–5.54)	5.18 (5.12–5.24)^a^	5.08 (5.00–5.17)^a^
hsCRP^c^	mg/L	0.56 (0.55–0.57)	0.46 (0.46–0.47)	1.39 (1.27–1.52)^a^	0.92 (0.81–1.05)^a^
Age-adjusted prevalence (95% CI), %					
AF		2.7% (2.4%–3.1%)	0.6% (0.5%–0.7%)	4.5% (3.3%–6.1%)^b^	1.4% (0.7%–2.8%)^b^
Overweight		34.3% (33.3%–35.3%)	37.2% (36.5%–38.0%)	10.6% (8.8%–12.7%)^b^	7.8% (5.7%–10.4%)^b^
Hypertension		46.0% (45.0%–47.1%)	42.2% (41.5%–43.0%)	84.9% (82.5%–87.0%)^b^	81.0% (77.3%–84.2%)^b^
Diabetes mellitus		8.8% (8.3%–9.4%)	5.4% (5.1%–5.8%)	34.1% (31.1%–37.1%)^b^	23.1% (19.6%–27.0%)^b^
Dyslipidemia		30.4% (29.5%–31.4%)	39.8% (39.0%–40.5%)	40.0% (36.9%–43.1%)^b^	29.2% (25.4%–33.3%)^b^
Current smoker		29.0% (28.0%–29.9%)	1.9% (1.7%–2.1%)	32.9% (29.9%–36.1%)	5.6% (4.0%–7.9%)^b^
Past smoker		31.3% (30.3%–32.2%)	1.6% (1.5%–1.8%)	38.7% (35.6%–41.8%)^b^	6.0% (4.2%–8.5%)^b^
Regular drinker		44.6% (43.6%–45.6%)	3.7% (3.4%–4.0%)	8.7% (7.0%–10.6%)^b^	2.2% (1.2%–3.9%)

AF, atrial fibrillation; ANCOVA, analysis of covariance; CI, confidence interval; BMI, body mass index; HDL-C, high-density lipoprotein cholesterol level; HbA1c, glycosylated hemoglobin; hsCRP, high-sensitivity C-reactive protein; SBP, systolic blood pressure; SD, standard deviation; TC, total cholesterol level.

Adjusted means were estimated using ANCOVA, and adjusted prevalences were estimated using logistic regression after adjusting for age (65 years).

^a^*P* < 0.05 compared to the adjusted mean in community dwellers by ANCOVA.

^b^*P* < 0.05 compared to the adjusted prevalence in community dwellers by logistic regression analysis.

^c^hsCRP data are expressed as age-adjusted geometric means (95% CI).

Table 2 shows the sex- and age-specific prevalences of AF for each age group of dialysis patients and community dwellers. The prevalences of AF were 3.8% in dialysis patients and 1.6% in community dwellers. SPRs of AF in dialysis patients compared to community dwellers were 2.53 (95% CI, 1.88–3.19) in all subjects, 1.80 (95% CI, 1.30–2.29) in male subjects, and 2.13 (95% CI, 0.66–3.61) in female subjects. The prevalence of AF increased with advancing age in both dialysis patients and community dwellers (from 0% in dialysis patients younger than 50 years to 10.6% in those aged 80 years or older, and from 0% in community dwellers younger than 30 years to 4.1% in those aged 80 years or older). The prevalence of AF in men was higher than in women in both dialysis patients (4.9% vs 1.6%; χ^2^ = 9.252, *P* = 0.002) and community dwellers (3.3% vs 0.6%; χ^2^ = 274, *P* < 0.001).

**Table 2.** Sex- and age-specific prevalences of AF in community dwellers and dialysis patients

Age group (years)	Community dwellers		Dialysis patients	
	
	*n*	AF (%)	*n*	AF (%)
Men and women				

20–29	251	0 (0.0%)	19	0 (0.0%)
30–39	834	1 (0.1%)	64	0 (0.0%)
40–44	1143	1 (0.1%)	68	0 (0.0%)
45–49	1650	6 (0.4%)	106	0 (0.0%)
50–54	2619	10 (0.4%)	187	1 (0.5%)
55–59	2918	16 (0.5%)	209	6 (2.9%)
60–64	4248	47 (1.1%)	218	10 (4.6%)
65–69	5128	90 (1.8%)	218	7 (3.2%)
70–74	4423	117 (2.6%)	199	12 (6.0%)
75–79	2443	90 (3.7%)	128	11 (8.6%)
≥80	797	33 (4.1%)	94	10 (10.6%)

Total	26 454	411 (1.6%)	1510	57 (3.8%)
			SPR	2.53 (1.88–3.19)

Men				

20–29	80	0 (0.0%)	15	0 (0.0%)
30–39	214	1 (0.5%)	45	0 (0.0%)
40–44	348	1 (0.3%)	45	0 (0.0%)
45–49	465	5 (1.1%)	70	0 (0.0%)
50–54	741	8 (1.1%)	113	1 (0.9%)
55–59	779	11 (1.4%)	142	4 (2.8%)
60–64	1368	39 (2.9%)	159	8 (5.0%)
65–69	1913	65 (3.4%)	145	7 (4.8%)
70–74	1802	92 (5.1%)	129	11 (8.5%)
75–79	1060	58 (5.5%)	77	10 (13.0%)
≥80	385	21 (5.5%)	63	8 (12.7%)

Total	9155	301 (3.3%)	1003	49 (4.9%)
			SPR	1.80 (1.30–2.29)

Women				

20–29	171	0 (0.0%)	4	0 (0.0%)
30–39	620	0 (0.0%)	19	0 (0.0%)
40–44	795	0 (0.0%)	23	0 (0.0%)
45–49	1185	1 (0.1%)	36	0 (0.0%)
50–54	1878	2 (0.1%)	74	0 (0.0%)
55–59	2139	5 (0.2%)	67	2 (3.0%)
60–64	2880	8 (0.3%)	59	2 (3.4%)
65–69	3215	25 (0.8%)	73	0 (0.0%)
70–74	2621	25 (1.0%)	70	1 (1.4%)
75–79	1383	32 (2.3%)	51	1 (2.0%)
≥80	412	12 (2.9%)	31	2 (6.5%)

Total	17 299	110 (0.6%)	507	8 (1.6%)
			SPR	2.13 (0.66–3.61)

AF, atrial fibrillation; SPR, standardized prevalence ratio.

Among individuals under 55 years of age, the age-specific prevalences of AF were less than 1% in both dialysis patients and community dwellers, with no differences between the two groups. Among individuals over 55 years of age, dialysis patients had 2.3-fold (in those 80 years or older) to 5.8-fold (in those 55 to 59 years) higher prevalences than community dwellers.

## DISCUSSION

We found that 3.8% of Japanese dialysis patients had AF and that the prevalence in dialysis patients was twice that in community dwellers living in the same area based on surveys conducted in 2003 to 2007. The prevalence rates were 4.9% in male dialysis patients and 1.6% in female dialysis patients. Both male and female dialysis patients had prevalence rates of AF twice those in community dwellers.

Two previous studies showed the prevalence of AF in Japanese dialysis patients. In 1996, Abe et al reported that there were 12 patients with AF among 221 dialysis patients in their sample, for a prevalence rate of 5.4%.^7^ The DOPPS study showed an identical prevalence rate (5.6%) using 219 cases of AF among 3935 dialysis patients recruited from 2002 to 2004.^8^ These prevalence rates are slightly higher than the prevalence in our study (3.8%). The KAREN study consisted of about 80% of dialysis patients in the whole study area, and 20% of the patients who did not provide informed consent were not included.^4^ Persons who did not participate in the survey were probably in poor condition and might have had heart disease, including AF. Thus, dialysis patients with AF might have preferentially been excluded in our study, and the prevalence of AF in our study might have been underestimated.

Several review articles are useful for comparison of the prevalence rates of AF in dialysis/ESRD patients in our study and those reported in previous studies. Zimmerman et al reviewed 20 studies regarding the prevalence of AF in dialysis/ESRD patients in Western countries, Taiwan, and Japan.^2^ The sample sizes ranged from 62 to 48 825, prevalence rates ranged from 2.8% to 26.7%, and the estimated mean prevalence rate was 11.6% based on pooled data for 223 477 subjects. The prevalence rate using pooled data was higher than that in our study and higher than the rates in the two previous studies based on Japanese dialysis patients.

Although no studies other than our study have shown the SPR of AF in dialysis patients compared to that in a representative general population, simple comparisons have suggested a 5- to 20-fold higher prevalence of AF in dialysis patients than in the general population in Western countries.^9^ Our results were identical to the prevalence of AF among ESRD patients in the USA in 1992, and only one-third of the prevalence in the United States in 2006.^9^ Winkelmayer et al explained why the prevalence of AF more than tripled from 1992 to 2006 in the United States, indicating that the sharp increase was due to predisposing factors, including older age, male gender, Caucasian race, and comorbid conditions. They proposed that older age and comorbid conditions (such as heart failure, peripheral artery diseases, coronary artery disease, and hypertension) might contribute to the increasing prevalence of AF.^9^ The reason for the lower prevalence of AF among Japanese dialysis patients compared to dialysis patients in Western countries might be the low prevalences of comorbid conditions, which are predisposing factors to AF, in Japanese dialysis patients.^8^

Several limitations of our study should be pointed out. As we mentioned earlier, persons who did not participate in the survey were probably in poor condition and might have had heart disease, including AF. The prevalence of AF was determined on the basis of routine examination of 12-lead ECGs recorded by each healthcare institute or dialysis institute, and some cases of paroxysmal AF might have been undiagnosed. These factors might have reduced the number of cases with AF in our study; therefore, the prevalence of AF might have been underestimated in the dialysis cohort or community dwellers, and the SPR of AF in dialysis patients might have been over- or under-estimated. The definition of AF in the IWATE KENCO Study was based on consensus by a trained clinical technician and a medical doctor in the Iwate Health Service Association, while the definition of AF in the dialysis cohort was based on consensus by three medical doctors. A stricter definition was used in the dialysis cohort study, and this might have contributed to over/underestimation of the SPR of AF in dialysis patients. The higher SPR of AF for all dialysis subjects (including male and female subjects) than that for men or women only was due to the imbalance of the male:female ratio in the reference group. A male:female ratio of 1.0 is desirable in the reference group; however, the ratio was 0.53 in our study, and this is one of the limitations.

Despite its limitations, our study provided sufficient evidence of a difference in the prevalence of AF between dialysis patients and community dwellers. Next, we should perform quantitative assessment of the risk for several outcomes attributable to AF among Japanese dialysis patients using a longitudinal study design.

## ONLINE ONLY MATERIAL

Abstract in Japanese.
