# Supplementary material for: Standardized Prevalence Ratios for Atrial Fibrillation in Adult Dialysis Patients in Japan
Source: J Epidemiol. 2016 May 5;26(5):272–6. doi: 10.2188/jea.JE20150077 (PMC4848326; doi:10.2188/jea.JE20150077)
Supplement: Abstract in Japanese. [file je-26-272-s001.pdf]

日本人成人透析患者の心房細動標準化有病比（Manuscript ID JE-2015-0077.）

大澤　正樹[1)]、丹野　高三[1)]、岡村　智教[2)]、米倉　佑貴[1)]、加藤　香廉[3)], 藤島洋介[4)]、小原　航[4)]、阿部　貴哉[4)]、板井　一好[5)]、小笠原　邦昭[6)], 大間々　真一[6)]、タンビル　チョウドリ　チュウリン[7)]、宮松　直美[8)]、石橋　靖宏[9)]、森野　禎浩[9)]、伊藤　智範[9)]、小野田　敏行[1)]、栗林　徹[10)]、蒔田　真司[9)]、吉田　雄樹[6)]、中村　元行[9)]、田中文隆[9)]、太田　睦子[11)]、坂田　清美[1)]、岡山　明[12)]

1) 岩手医科大学統合基礎講座衛生学公衆衛生学講座
2) 慶応大学医学部衛生学公衆衛生学講座
3) 岩手県立中央病院内科腎臓内科分野
4) 岩手医科大学医学部泌尿器科学講座
5) 盛岡大学栄養科学部栄養科学科
6) 岩手医科大学医学部脳神経外科学講座
7) カルガリー大学地域保健学部
8) 滋賀医科大学医学部臨床看護学講座
9) 岩手医科大学医学部内科学講座
10) 岩手大学教育学部保健体育科
11) 岩手県予防医学協会
12) 生活習慣病予防研究センター

抄録

背景　日本人透析患者の心房細動有病率は一般住民の心房細動有病率と比べて高いことが想定されるが、 実際には明らかにされていない。

方法　地域悉皆的コホート研究に参加した透析患者 1,510 名を解析対象として、同一地域に住む一般住民 26,454 名を基準とした透析患者の心房細動標準化有病比（SPR）を算出した。

結果 透析患者集団の心房細動有病率は 3.8% で一般集団では 1.6% だった。男性では心房細動有病率がそれぞれ 4.9% と 3.3%、女性では 1.6% と 0.6%だった。標準化有病比（95%信頼区間）は男女全体で 2.53 (1.88-3.19) 、男性で 1.80 (1.30-2.29) 、女性で 2.13 (0.66-3.61) だった。

結論 地域ベースの集団を対象とした解析結果では、日本人透析患者集団では一般集団に比べて心房細動有病率が 2 倍高かった。心房細動は心血管疾患死亡や罹患に強く影響するリスク要因であり、心房細動が日本人透析患者の予後にどのような影響を与えているのかを縦断研究で明らかにすることが望まれる。

KEYWORDS: 心房細動、末期腎不全患者、標準化有病比
